# Hypercalcemia due to Primary Hepatic Lymphoma

**DOI:** 10.1155/2016/1876901

**Published:** 2016-12-25

**Authors:** Andrew Hsu, Michael Gagnier, Elizabeth Ryer, Mohammed Salhab, Alan G. Rosmarin

**Affiliations:** ^1^Department of Medicine, University of Massachusetts Medical School, Worcester, MA, USA; ^2^Department of Hematology and Oncology, University of Massachusetts Medical School, Worcester, MA, USA

## Abstract

A 65-year-old female with a history of mixed connective tissue disease and pulmonary fibrosis on azathioprine, hydroxychloroquine, and prednisone (osteoporosis on teriparatide) presented with a 1-month history of hypercalcemia. After discontinuation of teriparatide, the patient's hypercalcemia persisted. Further evaluation revealed primary hepatic lymphoma as the source of her hypercalcemia.

## 1. Background

Hypercalcemia is a common complication of malignancy as described by Rodríguez-Gutiérrez et al. [1]. Typically, this is mediated by osteolytic metastases or secretion of parathyroid hormone related peptide (PTHrP). In both instances, calcitriol is typically suppressed in order to decrease intestinal absorption, renal reabsorption, and bone resorption of calcium. In some instances, hypercalcemia can be mediated by calcitriol. This occurs most commonly in the setting of sarcoidosis; however, it is reported to also occur in mycobacterium infections and hematologic malignancies [2]. Calcitriol-mediated hypercalcemia accounts for less than 1% of all malignancy-related hypercalcemia cases and it is usually found in non-Hodgkin's lymphoma (NHL), specifically diffuse large B-cell lymphoma (DLBCL), as was the case with our patient. Furthermore, this may be the first reported case of PHL potentially related to immunosuppressive therapy with hydroxychloroquine, azathioprine, and prednisone for mixed connective tissue disorder and pulmonary fibrosis.

## 2. Case Presentation

A 65-year-old female with a history of mixed connective tissue disease and pulmonary fibrosis on azathioprine, hydroxychloroquine, and prednisone (osteoporosis previously on teriparatide) presented with a 1-month history of hypercalcemia. The patient initially presented to her rheumatologist with complaints of weakness, dizziness, confusion, and difficulty with ambulation. Laboratory workup during that visit revealed hypercalcemia at 12.0 mg/dL (normal: 8.7–10.7 mg/dL) and albumin of 3.2 g/dL (normal: 3.5–4.8 g/dL). Her hypercalcemia was attributed her teriparatide use and this medication was discontinued; however, when her symptoms persisted, she presented to her primary care physician who found a calcium level of 13.6 mg/dL. She was admitted to the hospital where a skeletal survey was negative for lytic lesions and serum electrophoresis (SPEP) showed no monoclonal band. Further testing determined that she had PTH-independent hypercalcemia; parathyroid hormone (PTH) was decreased at 6 pg/mL (normal: 10–65 pg/mL), PTHrP was normal at 14 pg/mL (normal: 14–27 pg/mL), calcifediol (25-hydroxyvitamin D) was decreased at 18 ng/mL (deficiency < 20 ng/dL; insufficiency: 20–29 ng/mL; optimal ≥ 30 ng/dL), and calcitriol (1,25-dihydroxyvitamin D) was elevated at 159 pg/mL (normal: 18–72 pg/mL). Repeat calcitriol and PTH were 197 pg/mL and 6 pg/mL, respectively. The patient was rehydrated with intravenous (IV) fluids, and she received calcitonin, leading to improvement of her calcium to 10.7 mg/dL at the time of discharge. After discharge, she developed symptoms of fatigue, polydipsia, and polyuria. She presented to her primary care physician who found a calcium level of 15.6 mg/dL and was again admitted to the hospital. Upon further questioning, the patient did report a twenty-three-pound weight loss during the preceding two-month span but denied any fevers, chills, or drenching night sweats.

## 3. Investigation

Complete blood count revealed hemoglobin of 7.5 g/dL (normal: 12–16 g/dL), hematocrit of 22.4% (normal: 37–47%), and mean corpuscular volume of 104.9 fL (83–101 fL). Blood urea nitrogen was 21 mg/dL (normal: 7-23 mg/dL) and creatinine was 0.99 mg/dL (normal: 0.5–1.2 mg/dL). Serum iron, ferritin, transferrin, total iron binding capacity, transferrin saturation, folate, and vitamin B12 levels were all within normal limits. A peripheral blood smear was negative for any schistocytosis. Aspartate aminotransferase (AST), alanine aminotransferase (ALT), total bilirubin, and direct bilirubin were all within normal limits. Alpha-fetoprotein (AFP) and carcinoembryonic antigen (CEA) were within normal limits at 1.4 ng/mL (normal < 6.1 ng/mL) and 0.8 ng/mL (normal: 0–3 ng/mL in nonsmokers), respectively. Alkaline phosphatase (ALP) and lactic dehydrogenase (LDH) were elevated at 121 IU/L (normal: 30–115 IU/L) and 687 IU/L (normal: 110–240 IU/L), respectively. Hepatitis panel was negative for any evidence of a current infection or previous exposure.

**Figure 1:**
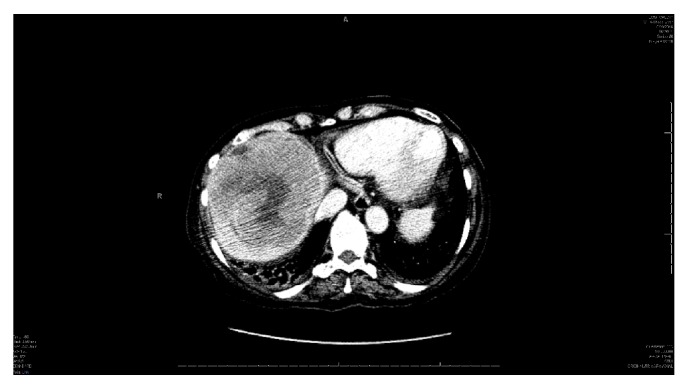
Axial view of primary hepatic lymphoma.

**Figure 2:**
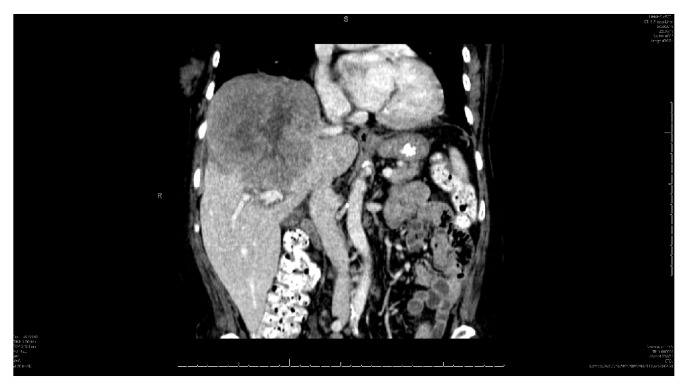
Coronal view of primary hepatic lymphoma.

Computed tomography (CT) of the chest did not reveal any lymphadenopathy or findings suggestive of granulomatous disease. CT of the abdomen and pelvis revealed an 11.3 × 10.7 × 11.1 cm mass in the right lobe of the liver with central areas of hypoenhancement likely reflecting necrosis (Figures 1 and 2). Another hypodense lesion, inferior to the previous mass, measuring 6 × 7 mm was also noted. Several mildly prominent pericaval, aortocaval, and pericaval lymph nodes were seen as well. Of note, no bony lesions were appreciated on either scan. A four-phase CT scan of the liver revealed no arterial enhancement of the mass, but the solid components demonstrated progressive delayed enhancement. Furthermore, there was mass effect on the right anterior portal vein and right hepatic vein without any vascular invasion. The smaller hypodense lesion inferior to the larger mass did not demonstrate enhancement which was compatible with findings of a cyst. Of note, CT of the head did not reveal any bony lesions or evidence of metastatic disease which was confirmed by magnetic resonance imaging (MRI).

Given the history concerning for malignancy coupled with the CT findings, the patient underwent a liver biopsy for tissue diagnosis of the lesion. Fine needle aspirate of the liver mass revealed malignant cells while cytology revealed CD45+, CD20+, CD10−, CD30−, BCL2−, BCL6+, and SOX10−. These findings were most consistent with a germinal cell center DLBCL.

## 4. Differential Diagnosis

The differential diagnosis of her PTH-independent hypercalcemia included teriparatide administration, milk-alkali syndrome, paraneoplastic syndrome, granulomatous disease, metastatic disease, and hematological malignancy, particularly lymphoma. On the lymphoma spectrum, those with non-Hodgkin's lymphoma [3–5], chronic myeloid leukemia (blast phase) [6], and adult T-cell leukemia-lymphoma may have PTHrP-induced hypercalcemia [7, 8]. Teriparatide administration was unlikely because the effects of hypercalcemia are transient, usually resolving sixteen hours after the last administration [9]. Milk-alkali syndrome was unlikely given that she reported the consumption of only six tablets over a span of a month. CT imaging was negative for any signs of granulomatous disease. Lastly, a bone scan, a skeletal survey, and CT imaging were negative for any lytic bone lesions which made metastatic disease as the cause of her hypercalcemia less likely.

Common differential diagnoses for a solitary hepatic lesion included hepatic cyst, hemangioma, focal nodular hyperplasia, hepatic adenoma, regenerative nodules, hepatocellular carcinoma, cholangiocarcinoma, or metastatic disease. Very rarely, a solitary hepatic lesion can be due to soft tissue sarcomas, hepatoblastoma, or non-Hodgkin's lymphoma (NHL).

## 5. Treatment

The patient was initially given prednisone 40 mg daily due to concerns of hypercalcemia secondary to granulomatous disease. This had been tapered down to prednisone 20 mg daily prior to the liver biopsy results. In addition, the patient was given IV fluids and given a single dose of zoledronic acid which led to an improvement of her hypercalcemia to 10.6 mg/dL. Upon diagnosis of her DLBCL, the patient was initiated on R-CHOP chemotherapy (rituximab, cyclophosphamide, doxorubicin, vincristine, and prednisone). At time of discharge, the patient's calcium level was 10.2 mg/dL and remained normalized at 9.8 mg/dL in her subsequent follow-up.

## 6. Outcome and Follow-Up

Given her age, elevated LDH, Eastern Cooperative Oncology Group (ECOG) performance status of two, and Ann Arbor stage IV disease, the patient's International Prognostic Index (IPI) was four, corresponding to 26% five-year survival [10]. The patient was initiated on her first cycle of chemotherapy during her hospitalization. Upon discharge, the patient and her family sought to transition her care to another tertiary hospital closer to her home.

## 7. Discussion

Hypercalcemia is a complex topic with many etiologies. From the furthest viewpoint, it can be categorized into PTH-dependent (PTHD) and PTH-independent (PTHI) causes. PTHD hypercalcemia can be suspected when elevated calcium levels are accompanied by normal to high normal PTH levels, suggesting inadequate suppression of PTH. The most common cause of PTHD is primary hyperparathyroidism (PHPT). A benign, single adenoma accounts for 80–85% of cases. Parathyroid gland hyperplasia including the multiple endocrine neoplasia (MEN) syndromes, hyperparathyroid jaw tumor syndrome, and familial isolated hyperparathyroidism is the culprit in 10–15% of cases. Less than 1% of the time, parathyroid malignancy is the etiology of PTHD hypercalcemia (Table 1) [3]. In our patient, PTH levels were adequately suppressed at 6 pg/mL which suggested that she did not have PTH-dependent hypercalcemia.

PTH-independent hypercalcemia has many etiologies. This includes solid tumors, hematologic malignancies, milk-alkali syndrome, medications, vitamin D intoxication, granulomatous disease, and calcitriol-mediated hypercalcemia.

Hypercalcemia mediated by PTHrP accounts for 80% of PTHI hypercalcemia cases secondary to malignancy. Osteolytic metastasis, often seen in multiple myeloma and breast cancer, is mediated by interleukin and accounts for 20% of malignancy-related hypercalcemia cases. In both of these scenarios, calcitriol is suppressed in order to decrease the intestinal absorption, renal reabsorption, and bone resorption of calcium. Less than 1% of malignancy-related hypercalcemia cases are mediated by calcitriol (Table 2) [2, 4].

Elevated calcium and calcitriol levels accompanied with decreased levels of PTH are highly suggestive of calcitriol-mediated disease. In the calcitriol-mediated hypercalcemia umbrella, sarcoidosis accounts for 49% of cases. Hematologic malignancies account for 17% of calcitriol-mediated hypercalcemia cases. Infection, solid organ malignancy, other granulomatous conditions, such as tuberculosis and systemic fungal infections, and idiopathic disease account for 8%, 5%, 4%, and 3% of cases, respectively (Table 3) [2]. Of the hematologic malignancies, the most common source of calcitriol-mediated hypercalcemia is non-Hodgkin's lymphoma, specifically DLBCL.

The mechanism of action of calcitriol-mediated hypercalcemia related to malignancy is unclear. Normally, 1-alpha hydroxylase is expressed in the kidneys and will convert inactive calcifediol to active calcitriol. It has been theorized that, in lymphomas, surrounding macrophages may have 1-alpha hydroxylase activity which contributes to the elevation of calcitriol levels. Hewison et al. demonstrated this phenomenon using immunolocalization [11].

Primary hepatic lymphoma accounts for 0.016% of all non-Hodgkin's lymphoma cases and 0.4% of primary extranodal NHL. The majority of primary hepatic lymphomas are found to be DLBCL [12]. Other pathologies are rare but have been reported in the literature. These include mucosa-associated lymphoid tissue (MALT), lymphoblastic, mantle cell, follicular, and Burkitt lymphomas [5].

Etiologic factors associated with PHL are human immunodeficiency virus (HIV), hepatitis B virus (HBV), hepatitis C virus (HCV), Epstein-Barr Virus (EBV), immunosuppressive therapy, and autoimmune diseases. Of note, HCV is reported in 40–60% of cases [6]. As far as immunosuppressive therapy is concerned, PHL has been well described in patients prescribed methotrexate for rheumatoid arthritis [13].

PHL more commonly occurs in the fifth decade of life and is three times more common in males [13]. Laboratory tests can help differentiate between hepatocellular carcinoma and PHL; ALP and LDH are typically elevated while AFP and CEA are within normal ranges as was the case in our patient [3, 6]. Radiographic imaging can help define the extent of disease; however, there are no characteristic findings that can clearly differentiate PHL from other differentials. Typically, imaging will show a solitary mass in 60% of PHL cases as seen in our patient (Figure 1). CT usually shows minimal to no enhancement on all phases likely due to the poor vascular supply. It is not uncommon to see a central hypodensity within the mass, commonly due to tissue necrosis [14]. Overall, there is no pathognomonic radiographic finding in PHL and therefore biopsy is considered the gold standard for the diagnosis of PHL. FNA should not be performed as the tissue may be necrotic and result in a false-negative result [6, 15].

Standard therapy for DLBCL is chemotherapy [6], particularly with the regimen of R-CHOP (rituximab, cyclophosphamide, doxorubicin, vincristine, and prednisone). Other treatment regimens with and without radiotherapy and surgery have also been described in the literature. For small localizing lesions, surgical resection may be sufficient as a stand-alone therapy, but this is not routinely practiced as relapse after surgery is common [5].

There are multiple observational and retrospective studies on survival rates for PHL. One study from the MD Anderson Cancer Center followed up 24 PHL cases for 20 years. Complete remission was achieved in 85% of cases and event-free 5-year survival rate was 70% [15].

## 8. Conclusion

Calcitriol-mediated hypercalcemia in malignancy is rare and accounts for less than 1% of all malignancy-related hypercalcemia cases. When present, it most commonly occurs in NHL, specifically DLBCL. PHL has known association with immunosuppressive therapy and autoimmune diseases and is well described in patients with rheumatoid arthritis prescribed methotrexate. To our knowledge, this is the first reported case of PHL potentially related to immunosuppressive therapy with hydroxychloroquine, azathioprine, and prednisone for mixed connective tissue disorder and pulmonary fibrosis.

## Figures and Tables

**Table 1:** PTH-dependent hypercalcemia etiologies and percent of occurrence. Parathyroid gland hyperplasia includes the multiple endocrine neoplasia (MEN) syndromes, hyperparathyroid jaw tumor syndrome, and familial isolated hyperparathyroidism. Rarely, primary parathyroid gland malignancy is the cause [3].

PTH-dependent hypercalcemia etiology	Prevalence
Single benign adenoma	80–85%
Parathyroid gland hyperplasia	10–15%
Parathyroid malignancy	Less than 1%

**Table 2:** The etiologies and frequency of PTH-independent hypercalcemia related to malignancy [2, 4].

Hypercalcemia due to malignancy etiology	Prevalence
PTHrP-mediated	80%
Osteolytic lesions	20%
Calcitriol-mediated	<1%

**Table 3:** Calcitriol-mediated causes of hypercalcemia and their prevalence. Information gathered from a review of 101 proven calcitriol-mediated hypercalcemia cases [2].

Calcitriol-mediated hypercalcemia etiology	Prevalence
Sarcoidosis	49%
Hematologic malignancy	17%
Infections	8%
Solid organ malignancy	5%
Other granulomatous conditions	4%
Idiopathic disease	3%
Diagnosis not made	14-15%
